# Perceptions of eye health in schools in Pakistan

**DOI:** 10.1186/1471-2415-6-8

**Published:** 2006-03-02

**Authors:** Khabir Ahmad, Mohammad Aman Khan, Mohammad Daud Khan, Mohammad Babar Qureshi, Tanveer Anjum Chaudhry, Clare Gilbert

**Affiliations:** 1 Pakistan Institute of Community Ophthalmology, Hayatabad Medical Complex, Hayatabad, Peshawar, Pakistan; 2 Section of Ophthalmology, Department of Surgery, Aga Khan University, Karachi, Pakistan; 3 International Centre for Eye Health, Clinical Research Unit, London School of Hygiene and Tropical Medicine, London, UK

## Abstract

**Background:**

Research exploring children's and their teachers' perceptions of eye health is lacking. This paper reports for the first time on perceptions of primary schoolchildren and their teachers of healthy and diseased eyes, things that keep eyes healthy and damage them, and what actions to be taken in case of an eye injury.

**Methods:**

Using *draw and write technique*, 160 boys and girls (9–12 years old) attending four primary schools in Abbottabad district, northern Pakistan, were invited to draw pictures in response to a set of semi-structured questions and then label them. Sixteen teachers who were currently teaching the selected students were interviewed one-on-one.

**Results:**

Analysis of text accompanying 800 drawings and of the interview scripts revealed that most children and teachers perceived healthy eyes to be those which could see well, and diseased eyes to be those which have redness, watering, dirty discharge, pain, and itching; or those which have "weak eyesight" and blindness. Among things that students and teachers thought damage the eyes included sun, television, and sharp pointed objects, particularly pencils. Teachers noted that children with eye problems "have difficulty seeing the blackboard well", "screw up their eyes", and "hold their books too close".

**Conclusion:**

We conclude that schoolchildren and their teachers had a good knowledge of eye health, but many of them had serious misconceptions e.g., use of kohl, medicines and eye drops keeps eyes healthy. Kohl is an important source of lead and can reduce children's intelligence even at low blood levels. Health education in schools must take into account children's existing knowledge of and misconceptions about various aspects of eye health. Such steps if taken could improve the relevance of eye health education to schoolchildren.

## Background

Refractive errors and trachoma have been identified as priority conditions for control within VISION 2020: The Right to Sight – a global initiative launched by the World Health Organization (WHO) and its partners to substantially reduce the burden of blindness and visual impairment by 2020 [1]. It has been advocated that screening for these conditions among children can be effectively performed by teachers [2-4].

There is growing body of evidence that many schoolchildren needing glasses do not get them because their refractive errors are not detected, despite concerns that uncorrected refractive errors might not only hamper children's physical, cognitive, and psychosocial development, but also future employability and earning opportunities [5].

In areas endemic for vitamin A deficiency and trachoma, the WHO also recommends early detection of these diseases in schools, and health education concerning their prevention and treatment [6]. Globally, an estimated 100 million children (less than 5 years of age) have vitamin A deficiency [7], and 150 million children have active trachoma. Almost 50 % of Pakistan's total population of around 154 million are children [8], but nationally representative data on childhood eye diseases are lacking. Pakistan has been classified by the WHO as a country with severe sub-clinical vitamin A deficiency in parts or whole of the country [9], and studies conducted in different areas of Pakistan show that 32–43% children under 5 have deficient serum vitamin A levels [10,11]. Trachoma is endemic in parts of the country [12].

A general lack of health care workers as health educators in Pakistan and many other developing countries supports the involvement of teachers in eye health education. However, there is a lack of literature on what they themselves know about common eye diseases, and their detection and management.

Schools also offer an excellent opportunity for health education about prevention of eye trauma which is a regular event among children and a major cause of blindness in one eye [13]. In addition, trauma management is highly specialized and expensive [6]. As in all health education strategies, the starting point should be what the target audience already knows and does, and what they need to learn. Much health education, however, lacks this foundation [14]. As Oakley *et al *argue: 'health education that builds on an accurate understanding of the beliefs and knowledge about health of the target group is probably more effective than strategies which lack this foundation. Much health education for children and young people has not been based on what they themselves know, believe, or want to know. There has been a tendency for children's voices, in particular, to be silent' [15]. This paper reports the findings of a qualitative study undertaken to explore primary schoolchildren's and their teachers' perceptions of eye health in Pakistan.

## Methods

The study involved 160 children (80 girls and 80 boys) aged 9–12 years and their 16 teachers (8 men, 8 women) in four primary schools (two private, two government) in the Abbottabad district, northern Pakistan. In government-run schools the education is almost free, attracting children from lower socio-economic groups. Private schools generally cater for children from rich families. Two schools (one for boys and one for girls) were selected from the 2 categories, based on easy accessibility. Permission to conduct the study was obtained from the Ethics Review Committee of Pakistan Institute of Community Ophthalmology, the District Education Officers (DEO) for government schools, and head teachers of each school. Informed verbal consent was obtained from children. Children aged 9–12 years were selected because we assumed children below this age might not be able to understand how to use the draw and write technique. In each school, a list of all children aged 9–12 years was developed using the attendance register and then a sample of 40 children was selected by the principal investigator (KA). Teachers or school administrators had no role in the selection of children.

Although there are a range of methods to elicit qualitative information from children, a technique that has become popular is "draw and write" [16], which was first described by Williams in 1989 [17]. This method has been used to explore children's views about a range of health topics, including HIV/AIDS, drugs, and skin cancer [14,18-20]. We used it to explore primary schoolchildren's perceptions of healthy and diseased eyes, things that keep eyes healthy and damage them, and what action to take in case of an eye injury. In additions, we interviewed teachers one-on-one to explore their perceptions of eye health i.e. how to detect common eye problems in children, what advice to give to children to keep their eyes healthy and what actions to take if something happens to a child's eyes.

The questions initiating draw and write exercise were: What are the characteristics of a healthy eye? What do diseased eyes look like? What are the things that damage the eyes? What would you do to keep your eyes healthy? What action (s) will you take if an accident injures your eye? These questions had been pre-tested in a group of children in Charsadda district.

The exercise was undertaken in classrooms, so that it appeared as an extension of normal classes. The draw and write technique was explained to them, and drawing material distributed. Each question in turn was written on the blackboard in Urdu and English in government and private schools, respectively. Children were asked to respond to the question by drawing, and then labeling or describing it in the language they felt comfortable in. Any child unable to write told the facilitator what they wanted to write who then wrote their views down verbatim. Children who did not understand the exercise were given instructions in Hindko, Urdu, and Pakhtu languages as appropriate. All the sheets were collected before the next question was asked.

The questionnaire used to interview teachers was a modified version of the one used for children, and which had been piloted in a school in Charsadda district. It had three additional questions: How would you detect common eye problems in children? What would you advise children to keep their eyes healthy? What would you do if something happens to a child's eyes? All interviews were conducted in Urdu. In each school, we selected 4 teachers who currently taught the selected children. One of the investigators (KA), who had previous experience in conducting interviews and who received additional training for the study, interviewed teachers one-on-one in their schools. The interviewer encouraged respondents' participation by giving prompting questions: "Tell us more....," "Keep talking", "And..." Notes were taken during interviews, each of which lasted 30–45 minutes.

Children provided 800 drawings, and their accompanying text. Colloquial terms used by the children were translated in the classroom as soon as the exercise was completed. Labels were translated from Urdu into English. To avoid subjectivity, only labels and not drawings were assessed. The interview scripts were translated into English. Each script was read to get general and specific ideas. Similar themes from all interviews and labels were grouped and then organized into major categories and subcategories. Supporting quotations were selected to illustrate the main or atypical themes. Quantitative data were analysd using SPSS 10.0.

## Results

### Perceptions of healthy eyes

**Figure 1:**
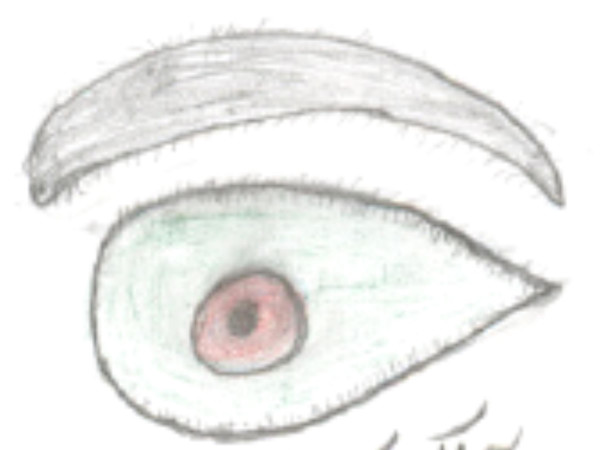
Drawing of healthy eyes by a girl from the government school. "Eye are to see with. We can't see without eyes. Eyes are a great blessing of God. Healthy eyes have vision. Eyes have three colours."

**Figure 2:**
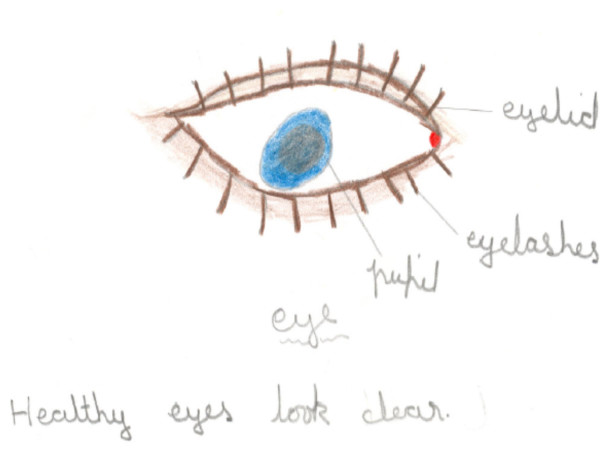
Drawing of healthy eyes by a girl from the private school.

Most children (Table 1; Figure 1 and 2) and teachers (also Table 1) perceived healthy eyes to be those which can see well or which are beautiful, bright, fresh-looking, and neat/clean. One student mentioned 'full of dignity' as a characteristic of healthy eyes. A small number of teachers believed that health eyes are those which are "not squinting". One teacher said that "they should be straight." Some teachers believed healthy eyes were those which do not have any signs and symptoms of disease such as discharge, watering, pain, and burning.

**Table 1:** Children's and their teachers' perceptions of healthy eyes.

	Children† (n = 160)		Teachers (n = 16)	
Characteristics*	*Frequency*	*%*	*Frequency*	*%*

**Can see well**	120	75.0	12	75.0
**Characteristics related to anatomy of eye**				
Contain white	18	11.3	...	...
Contain black	12	7.5	...	...
Have got pupil	12	7.5	...	...
Have got eyelids	9	5.6	...	...
Have got eyelashes	5	3.1	...	...
**Characteristics related to good appearance**				
Are beautiful	15	9.4	...	...
Are big	7	4.4	...	...
Are bright	5	3.1	5	31.3
Are clean/neat	5	3.1	3	18.8
Are fresh-looking	...	...	4	25.0
**Allah's blessing**	20	12.5	...	...
**Absence of disease symptoms(e.g. watering, discharge, squint)**	10	6.3	5	31.3

*Respondents could mention more than one characteristic of healthy eyes.

†Only characteristics mentioned by at least 3 % students included in the table

"Healthy eyes are bright. They are neither too big nor too small. They should have good vision." [Male teacher: private school]

"Healthy eyes are neither yellow nor red. Healthy eyes [do not] water or discharge. They should be clean and bright." [Female teacher: government school]

"Healthy eyes can see well. Eyes have three colours. Eyes are to see with. Eyes are a great blessing of God. Without eyes, one can't see anything." [Girl: government school]

"Healthy eyes enable human beings to see things well. A healthy eye is clean." [Boy: private school]

### Perceptions of diseased eyes

**Figure 3:**
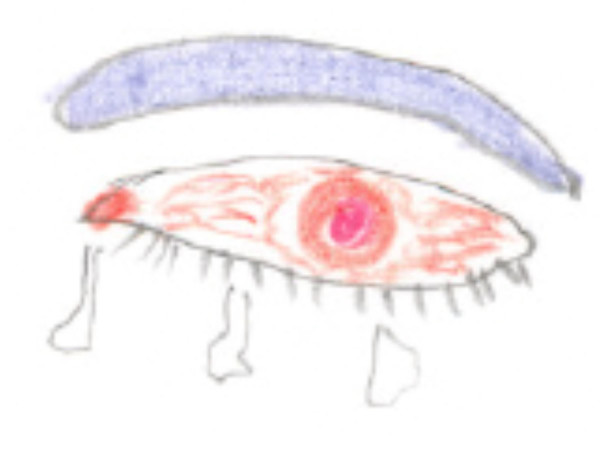
Drawing of diseased eyes by a boy from private school. "My eyes are diseased. They remain red all the times. And that is why they water."

**Figure 4:**
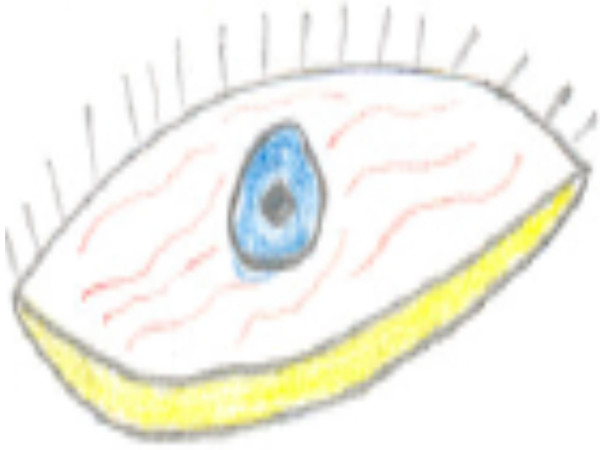
Drawing of diseased eyes by a boy from private school. "We can't see well with a diseased eye. A diseased eye is painful."

**Figure 5:**
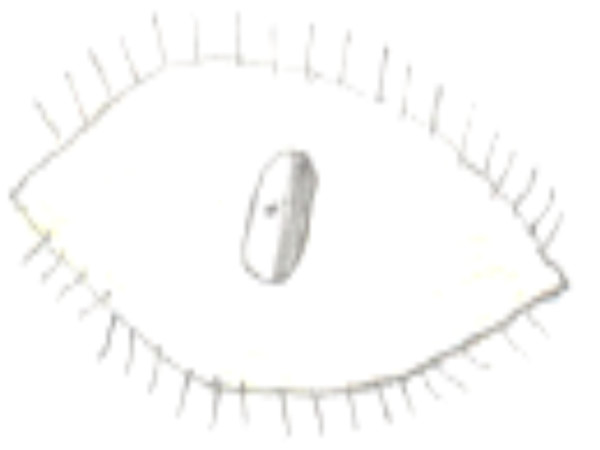
Drawing of diseased eyes by a boy from government school. "This eye has cataract."

Two main themes emerged from children's and teachers' responses about characteristics of diseased eyes: they were a) red/painful eye, and b) eyesight problems. Most children (Table 2; Figure 3, 4 and 5) and teachers (also Table 2) also said diseased eyes watered, had dirty discharge, or itched. They also believed that diseased eyes were those that can not see well, are blind or have cataract. Five (37.6%) teachers said that diseased eyes squinted.

**Table 2:** Children's and their teachers' perceptions of diseased eyes

	Children† (n = 160)		Teachers (n = 16)	
Characteristics*	*Frequency*	*%*	*Frequency*	*%*

**Signs and symptoms of red eye**				
Are red	66	41.3	12	75.0
Have watering	25	15.6	7	43.8
Have dirty discharge	21	13.1	3	18.8
Are painful	10	6.3	1	6.3
Have allergy	10	6.3	...	...
Have bleeding	9	5.6	...	...
Have itching/burning	6	3.8	4	25.0
Have kokray(trachoma)	...	...	3	18.8
Are swollen	...	...	4	25.0
**Signs and symptoms of eyesight problems**				
Can't see well/have weak eyesight	46	28.8	8	50.0
Are blind/can't see at all	23	14.4	3	18.8
Have cataract	23	14.4	7	43.8
**Signs and symptoms related to size and shape**				
Have growth	16	10.0	...	...
Are squinting	...	...	5	31.3
One eye is smaller than the other	...	...	3	18.8
**Appearance**				
Are bad-looking	7	4.4	1	6.3

*Respondents could mention more than one characteristic of diseased eyes.

†Only characteristics mentioned by at least 3 % students included in the table

"Diseased eyes are red and have blurred vision. They itch. Diseased eyes have cataract. They have poor vision. There is headache due to weak eyesight." [Female teacher: private school]

"The colour of diseased eyes is a little different from that of normal eyes. Diseased eyes are red, and can not see well. They are painful too. They often water." [Boy: government school]

"A diseased eye has weak eyesight. A diseased eye is sometimes red, due to which you have to itch it, and it does not look clean." [Girl: private school]

"When the eyes get diseased, the white of the eye becomes red. There are quick jerky movements. They are not straight. The lens sometimes comes out." [Male teacher: private school]

### Things that damage the eyes

**Figure 6:**
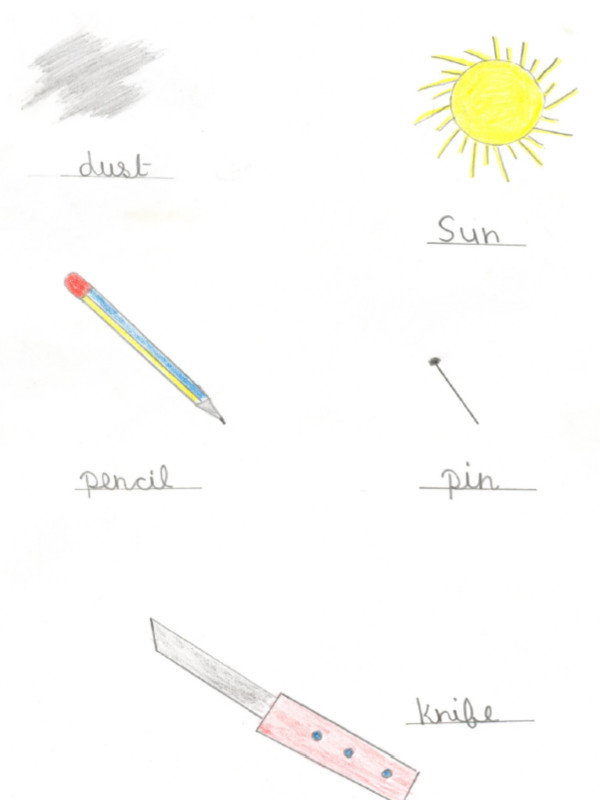
Drawing, by a girl from the private school, of things that damage the eyes.

The strongest message from teachers and children was their concerns about the adverse effects of very bright light, sunlight, watching television for too long or sitting too close to the television, and of chemicals and diet. A large number of teachers (Table 3) as well as children (also Table 3; Figure 6) perceived sharp pointed objects (such as pencils, sticks, stones, needles, wood, pens, and scissors) to be eye-damaging. Fourteen (8.8%) students believed that books/book-reading were not good for eyes while 11 (6.9%) students reported "too much book-reading" was harmful. Many students implicated eating chilies, onion, eggs and beef in causing eye damage.

**Table 3:** Children's and their teachers' perceptions of things that can damage the eyes

	Children† (n = 160)		Teachers (n = 16)	
Factors*	*Frequency*	*%*	*Frequency*	*%*

**Factors related to light**				
Sun	115	71.9	7	43.8
Television use (e.g. too long or too close or while lying down or with concentration).	61	38.1	7	43.8
Bulb	14	8.8	2	12.5
Bright light	11	6.9	7	43.8
Dim light	5	3.1	4	25.0
**Sharp pointed objects**				
Pencil	33	20.6	1	6.3
Stick	14	8.8	1	6.3
Stone	10	6.3	2	12.5
Needle	9	5.6	1	6.3
Wood	6	3.8	1	6.3
Pin	5	3.1	1	6.3
**Habits**				
Reading in dim light or in bright light	14	8.8	9	56.3
Too much book reading	11	6.9	2	12.5
Reading too close			6	37.5
Reading while lying down			3	18.8
**Diet**				
Chillies	26	16.3	...	...
Onion	7	4.4	...	...
**Chemicals**				
Soap	22	13.8	1	6.3
Tear gas	6	3.8	...	...
**Pollution**				
Dust including chalk dust	29	18.1	7	43.8
Smoke	6	3.8	4	25.0

* Respondents could mention more than one thing.

†Only characteristics mentioned by at least 3 % students included in the table

"Looking at the sun damages the eyes. Dim light damages our eyes. Chilies cause eye damage." [Boy: government school]

"Things that can be damaging are: TV, video games, films, cartoons, etc. If children throw stones, pencil and pen into your eyes, it can be dangerous. If you watch TV for 2–20 hours it is harmful to your eyes." [Girl: private school]

"Chalk dust, excessive TV watching and insect bite damage the eyes." [Female teacher: private school]

"Watching TV too close, say from around 10 feet, damage the eyes because TV emits rays. An accident can also damage the eyes. Quite often stones, pointed objects, nail and sharp light damage the eyes of children." [Female teacher: private school]

"Intense light, smoke, dust and dirt, reading book while in a moving vehicle, taking ice-cream and hot tea and curry – all these things damage the eyes." [Male teacher: private school]

### How to detect common eye problems in children

As shown in Table 4, most teachers said children with common eye problems have difficulty seeing the blackboard and nearly half (7 out of 16) said these children hold their books too close. One teacher said such children often screw up their eyes when reading. Most teachers reported other signs and symptoms such as redness, watering, rubbing, discharge, swelling, itching, and pain.

**Table 4:** How teachers would recognise common eye problems in children

Eye problems *	Frequency (n = 16)	%
**Problems related to vision**		
Have difficulty seeing the blackboard	12	75.0
Hold books too close	7	43.8
Have difficulty watching TV or reading book	2	12.5
Others (e.g. cannot read books in dim light; are colour blind)	6	37.5
**Red painful eye**		
Have redness	11	68.8
Have watering	9	56.3
Rub their eyes	7	43.8
Have discharge in their eyes	3	18.8
Have swollen eyes	3	18.8
Have burning sensation in eyes	2	12.5
Have pain in their eye; trachoma or stick eye	3	18.8
**Other signs and symptoms**		
Have yellow eyes	7	43.8
Have black circles around eyes	2	12.5
Are physically weak	2	12.5
Others (e.g. eyes don't look fresh; drowsy; blink too often)	13	81.3

*Respondents could mention more than one eye problem.

"He cannot see the blackboard clearly. [They] cannot read the writing on the blackboard from back benches. [So] we ask them to sit close to the blackboard. Eyes of such children water. They cannot read when the light deteriorates. They have pain in their eyes. Diseased eyes are red or yellow." [Male teacher: private school]

"They wear thick glasses. Their eyes start burning when they watch TV." [Female teacher: government school]

"Some of them come to us and tell us they cannot see the blackboard, TV and books." [Female teacher: private school]

### Recipe for healthy eyes

To keep their eyes healthy most teachers would advise students to "wash their faces with clean water", and avoid sunlight, bright light, self-medication, reading in dim light, touching/rubbing eyes with dirty hands, and "holding the book too close" (Table 5). Some teachers said that they would advise children to eat vegetables (especially carrots), and to drink milk, and one teacher recommended vitamin A.

**Table 5:** How teachers would advise children to keep their eyes healthy

Type of advice*	Frequency (n = 16)	*%*
**Advice related to cleanliness**		
Wash your face with clean water, and keep eyes clean	8	50.0
Splash water into your eyes	3	18.8
Avoid touching /rubbing eyes with dirty hands	2	12.5
**Advice related to food**		
Foods to eat (e.g. vegetables, carrots, milk, cucumber, turnip, salad)	12	75.0
Foods to avoid: spicy, citrus, very hot food	3	18.8
Take balanced diet, use proteins	2	12.5
Take vitamin A	1	6.3
**Advice related to medication**		
Contact doctor if there is an eye problem	2	12.5
Avoid self-medication, use eye drops as advised by doctor	3	18.8
Use glasses if advised	1	6.3
Use only time-tested kohl- the white kohl	1	6.3
Check eyesight every 6 months	1	6.3
Avoid lotions, chemicals, and beautifiers unless indicated	1	6.3
Avoid use of kohl	1	6.3
**Advice related to light and heat**		
Avoid sunlight, use sunglasses	10	62.5
Television use (e.g. not too long or too close or while lying down).	5	31.3
Do not take a walk in cold wind	1	6.3
**Advice related to reading**		
Avoid reading in dim light or in bright light	9	56.3
Do not hold book too close	5	31.3
Do not read while lying in bed	2	12.5
Read in sufficient light	2	12.5
Do not read in a moving vehicle	1	6.3
If you cannot see the blackboard well, please contact me	1	6.3
**Advice related to environment**		
Avoid dust and smoke	4	25.0
See greenery, or take a walk on green grass	3	18.8
Plant trees	1	6.3
**Advice related to injury prevention**		
Avoid playing with pen/pencil	1	6.3
Do not hit each other with stone	1	6.3
Do not slap each other	1	6.3
Avoid rubbing eyes	1	6.3
Avoid striking head against hard objects	1	6.3
Avoid jumping down from a height	1	6.3
**Advice related to other things**		
Do not use colour papers. Use only white or red papers	1	6.3
Get enough sleep	1	6.3
Tell your father if there is any eye problem	1	6.3

*Respondents could give more than one advice

"I will ask them to take vegetables such as spinach, avoid dust and pollution, (and) live in clean environment. The classroom should have good ventilation and a good blackboard." [Male teacher: private school]

But teachers had a number of misconceptions: some teachers said they would advise them to "splash water into your eyes", "see greenery", "use white kohl", or avoid "citreous and spicy food".

"Keep your eyes clean in all sorts of conditions. Wash your eyes, when they are open, with clean water. Wash it three to four times a day. Do not look at something constantly for too long. When reading, the light should not come into your eyes, but it should fall on the book. Keep an appropriate distance between the book and eyes. Ideally it should be one and a half feet. Never look directly at the sun. Avoid reading book in dim light, in candle light and in lantern light. Do not use colour papers for writing. They will damage the eyesight. Only use white papers. However, very young children can use red papers. To keep eyes healthy walk barefooted in the morning dew. This should be done for 30–45 minutes. See greenery for 15–20 minutes a day." [Female teacher: government school]

**Figure 7:**
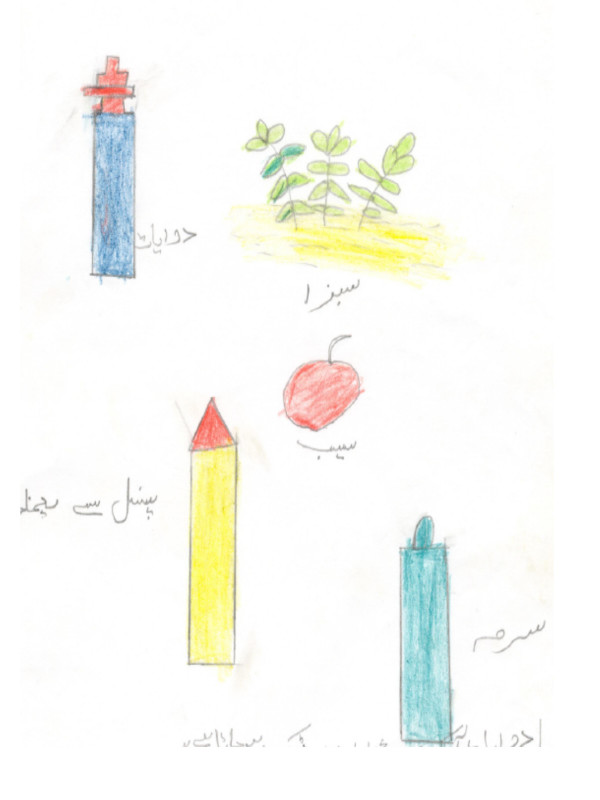
Drawing, by a girl from the government school, of things that keep eyes healthy. "Eyes can be kept healthy by putting medicines and kohl in eyes; looking at greenery, eating apple, and avoiding pencil."

**Figure 8:**
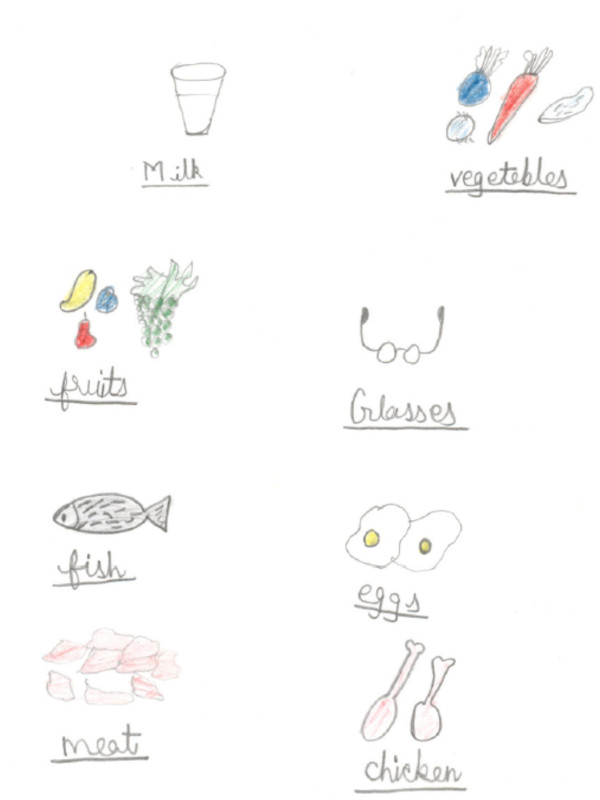
Drawing, by a girl from the private school, of things that keep eyes healthy.

By contrast, children noted they would wear glasses (53/160 [33.1%]), use medicines (30/160 [18.8%]), and visit a doctor (8/160 [5.0%]) to keep their eyes healthy, and more than a third (56/160 or 35.0%) indicated they would eat fruits, vegetables and nuts (Table 6; Figure 7 and 8). Twenty six (16.3%) students mentioned they will wash their faces, and 13 (8.1%) would avoid books/book-reading to keep their eyes healthy. Over a quarter of students (41/160) pointed out they would use home remedies/traditional medicines such as ice packs, kohl, and rose water to keep their eyes healthy. Sixteen (10.0%) students would avoid playing with sharp/pointed objects. They also said they would "see greenery".

**Table 6:** Children's perceptions of what they will do to keep their eyes healthy

Actions*	Frequency† (n = 160)	%
**Glasses and drugs**		
Wear glasses	53	33.1
Use medicine	30	18.8
Visit doctor	8	5.0
Put eye drops	7	4.4
**Sources of light**		
Avoid Sun	42	26.3
Avoid TV, or sitting close to TV	29	18.1
Avoid light bulbs, candle light	12	7.5
**Environment**		
See greenery	40	25.0
Avoid chalk	16	10.0
Avoid dirt	8	5.0
**Diet**		
Eat Fruits	34	21.3
Eat apple	28	17.5
Eat grapes	7	4.4
Eat mango	6	3.8
Drink milk	16	10.0
Eat eggs and meat	11	6.9
Eat vegetables	26	16.3
Avoid eating chillies	11	6.9
Eat carrots and other vegetables	17	10.6
Take almond	11	6.9
**Habits**		
Do face-washing	26	16.3
Avoid books/book-reading	13	8.1
**Traditional medicine/home remedies**		
Put ice packs on eye	13	8.1
Use kohl	26	16.3
Put rose water (arq-e-gulab) in eyes	5	3.1
Avoid putting pepper in eyes	5	3.1
**Sharp objects**		
Avoid playing with pencils	14	8.8
Others	7	4.4

* Respondents could mention more than one action.

†Only characteristics mentioned by at least 3 % students included in the table.

"Seeing greenery keeps the eyes healthy. Putting drops into eyes keep them healthy. Taking apple sharpens the eyesight. One should never sit close to TV or watch it in darkness. One should not read books in sharp light. The use of kohl keeps the eyes well. We should protect our eyes from pencils." [Girl: government school]

### Actions in case of an eye injury/problem

**Figure 9:**
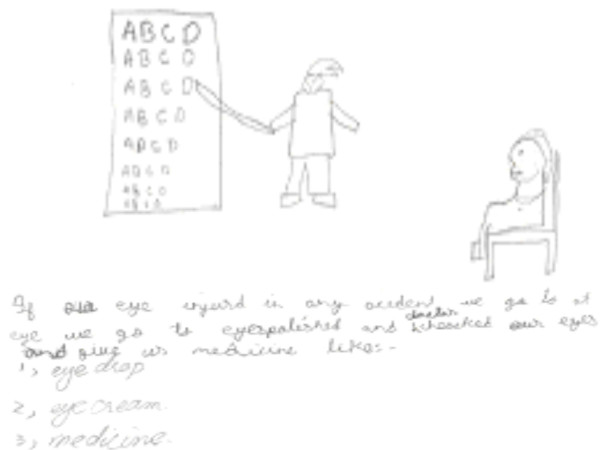
Drawing, by a boy from the private school, of action to take in case of an eye injury.

The majority of children (56.3 %) indicated that they would "consult a doctor" if they injured their eyes (Figure 9).

"When our eyes get injured we will go to a doctor and act upon his advice, and will use the prescribed medicines." [Boy: government school]

Seven students would put kohl in their eyes after injury while two would instill chilies. Sixty four (40.0 %) children said they would use medicines, such as ointment (50 children; 31.3 %) or eye drops (37 children; 23.1%), and 38 (23.8%) would apply a bandage. Ten (6.3%) students mentioned they would undergo an operation. Only one student mentioned she would get her vision checked by a doctor in case of an eye trauma. Teachers unanimously reported that they would manage "minor eye problems", such as foreign bodies, redness, burning, and watering, but would refer "serious" and "more severe" problems to a doctor.

"If anything goes into a child's eyes, I'll ask him to avoid rubbing [them] because it may cause an injury. If the foreign body can be removed, we will remove it. If not, we'll contact a doctor. If there is burning, we will put ice packs on the eyes. This will reduce burning and redness. If there is dirty discharge, we'll clean his eyes with a handkerchief. If the problem is serious, then a doctor will be contacted." [Male teacher: private school]

"If something falls into eyes, we'll wash it. We will use eye drops and *Polyfax *eye ointment. We'll ask them to close them for sometime. If there is irritation due to light, we will ask her to stay in the dark. If there is redness, pain or loss of vision, a doctor would be contacted." [Female teacher: government school]

## Discussion

To our knowledge this is the first study to explore the perceptions of primary schoolchildren and their teachers in relation to eye health. This is also the first study to use a novel technique called Draw and Write to find out about what kids and teachers know and do about eye disease. The results provide important insights into the understandings of common eye diseases among children in Pakistan where health education is not an integral component of primary schools curriculum. Although children in primary schools are not taught about eyes, most children as well as their teachers had a good knowledge of various characteristics of healthy eyes, including seeing well and giving good cosmetic appearance. However, they also need to know that healthy eyes are those in which the eyelids open and close properly, the white is white, the cornea is clear, and the pupil gets smaller in bright light [21]. In our study children's knowledge of the anatomy of the eye was poor, which may be because children are not taught this. However, guidelines on health education in schools, issued by the WHO, UNICEF and UNESCO recommend that children at this level be taught about the following parts of the eye: conjunctiva, cornea, pupil, iris and lens[22], but they make no mention of eyelids, which protect the eyeball and help lubricate the eye surface. One way children could be taught parts of the eye is to ask them, ideally in their art class, to look at each other's eyes and draw what they see. When finished, they could be asked to label what they have drawn. Once they recognize different parts of eye, children could be taught various functions of the eye.

Students as well as teachers reported irritable/red/painful eyes and loss of vision as the two most important characteristics of diseased eyes. The leading cause of red eyes in Pakistan is viral conjunctivitis (which gets better on its own). Trachoma is endemic in parts of the country [12] where it is an important cause of irritable, red eyes. Teachers as well children can play a vital role in controlling their known risk factor: eye-hand contact, flies, and handkerchiefs- and towel-sharing. Health education in schools also can play an important role in reducing the burden of trachoma by promoting face washing because dirty faces can help spread the disease from person to person through eye-seeking flies or contaminated fingers. Learning about environmental improvements to change living conditions for the better, including fly control and sanitation is also necessary. In much of Pakistan, people keep their livestock in their houses and awareness of domestic waste management is lacking. Such practices needs to be discouraged [23-26].

Similarly, students needs to be taught about uncorrected refractive errors because they adversely affect academic performance, increase the risk of trauma, and result in social isolation and stigma [4,5,27]. It was encouraging to note that teachers had a good understanding of how they would detect such problems, but they were not aware of other symptoms and signs of uncorrected refractive errors such as short attention span, difficulty writing in straight lines, headache, and low self-esteem. Developing countries, including Pakistan, lack sufficient primary eye care workers to screen for refractive errors. In these settings, teachers if equipped with necessary knowledge and skills, can play a vital role in reducing the burden of uncorrected refractive errors [3,28].

It was interesting to note that the majority of the students and teachers perceived the sun, watching television, and other sources of light to be eye-damaging. In some respects they are correct, as sun is a common source of ultraviolet radiation, which has been implicated as a risk factor for some age related eye conditions [29,30], while prolonged staring at the sun can cause macular burns [31-33]. Sharp pointed objects were reported as potentially damaging to the eyes, but none mentioned sports, fireworks, playing with discarded syringes or hazardous toys, which are among the commonest causes of eye injuries among schoolchildren [34-37]. Therefore health education programmes for schoolchildren must focus on these important causes of eye injuries. It was also interesting to note that many students as well as teachers felt that prolonged close-up work, such as reading, damages the eyes. It is argued that prolonged reading may contribute to the development of myopia (shortsightedness) which is an important public health problem in several East Asian countries [38,39].

A very positive message from teachers as well as children is that many of them recognize the need to eat fruit and vegetables to promote eye health. Vitamin A deficiency is a significant public health problem in Pakistan and school health education about it is essential, particularly for girls, who need to be made aware of daily vitamin A requirements, and of foods rich in vitamin A (e.g. breast milk, liver, eggs, yellow fruits and dark green leafy vegetables), so that they will be able to provide their future children with a healthy diet. Other very positive messages from both groups are that many of them recognize the need to wash faces, avoid sharp pointed objects and dust, and visit a doctor.

Students and teachers had many misconceptions concerning how eyes can be kept healthy. A third of the students reported that eye drops and ointment keep their eyes healthy. But the unnecessary use of medicine is potentially harmful: for example, topical steroids can cause cataract and glaucoma. Twenty six (16.3%) children said they would use kohl to keep their eyes healthy. Although kohl makes eyes look beautiful, it can cause lead poisoning [40-43]. Several studies have shown that high levels of lead exposure leads to anemia, kidney diseases, and neurological disorders, and even low levels can impair children's intelligence[44,45]. Advising children to "splash water into your eyes" "avoid spicy or citreous food", or instill rose water could adversely affect their eyes because these are harmful practices. For example, avoidance of citreous fruits, hot spicy and bitter food and chilies may lead to vitamin and mineral deficiencies and thus ocular morbidity. Other misconceptions are merely traditional practices that neither harm nor help, but they may be psychologically comforting, e.g., seeing greenery. The only problem is that misconceptions stand in the way of real information. It is interesting to note that the options provided by the students to keep their eyes healthy, in a way, may reflect what their parents may think and what is generally done/followed in the society. For example, use of kohl in Pakistan is so common that it is now part of culture/tradition as are use of rose water and eye drops without prescription.

Students' responses to the question about eye injury are equally significant as 56.3% of them believed they would contact a doctor if they injured their eye. Any injury of the eye should be considered a medical emergency because immediate medical care by an ophthalmologist is more likely to improve prognosis than delayed care. Many students mentioned they would use medicine, ointment or eye drops, but self-medication can do more harm than good, and may lead to delays in seeking appropriate medical care. By contrast, teachers said they would manage minor eye problems themselves, but refer "severe" and "more serious problems" to a doctor. Teachers would use "rose water, water splashes, and icepacks to treat red eyes; kohl to treat minor injury; and hot fomentation for foreign bodies. But some of these are harmful eye practices and should be avoided. In addition, teachers do not have sufficient knowledge or skills to always be able to distinguish minor injuries from major.

Our study had the following limitations: the findings may not be generalisable because schools participating in the study were not randomly selected. Second, our use of draw and write technique was based on assumptions that drawing enables children to communicate ideas better than conversational language, which may not be the case. Critics of the draw and write technique also argue that the technique has the potential for adult researchers to place their own interpretation on the drawing and words of children, that drawings by children are not the direct translations of their mental states, and that the technique may not create the potential for children to have their ideas heard and understood by researchers[16].

Our study provides baseline data on school children's and their teachers' perceptions of eye health and could be used to design further research as well school eye health education programme in Pakistan and other developing countries. Both groups had a good knowledge of eye health, but their misconceptions could adversely affect not only children's eye health but also intellectual development. Health education for children must take into account their existing knowledge of and misconceptions about various aspects of eye health. Such steps, if taken, could improve the relevance of eye health education to school children. We also suggest that primary school children need to learn how to draw and label an eye, steps to prevent/control common childhood conditions such as vitamin A deficiency, trachoma, trauma and refractive errors; how to make and use a vision chart and identify individuals with visual impairment in school and elsewhere; and what can be done to help those with vision problems. Such learning would be more meaningful if children are engaged in the learning process because most children find passive learning situations such as traditional talk-and-chalk sessions less interesting than action-oriented learning. However, constraints such as lack of resources and teachers' training should be addressed.

## Competing interests

The author(s) declare that they have no competing interests.

## Authors' contributions

CG and KA conceived the report. KA collected the data, performed the statistical analysis, and drafted the manuscript. MAK, MDK, MBQ, TAC and CG contributed to review, and to the revision of the report. All authors read and approved the final manuscript.

## Pre-publication history

The pre-publication history for this paper can be accessed here:



## References

[B1] WHO (2005). VISION 2020: The Right to Sight. www.v2020.org.

[B2] Foster A, Gilbert C (1997). Epidemiology of visual impairment in children. In: Taylor D, ed. Paediatric ophthalmology., .. London:.

[B3] Limburg H, Kansara HT, d'Souza S (1999). Results of school eye screening of 5.4 million children in India--a five-year follow-up study. Acta Ophthalmol Scand.

[B4] Wedner SH, Ross DA, Todd J, Anemona A, Balira R, Foster A (2002). Myopia in secondary school students in Mwanza City, Tanzania: the need for a national screening programme. Br J Ophthalmol.

[B5] Dandona R, Dandona L (2001). Refractive error blindness. Bull World Health Organ.

[B6] WHO (1997). Strategies for the prevention of blindness in national programmes: a primary health care approach , 2nd edit. Geneva, World Health Organization,.

[B7] Unicef (2005). Unicef statistics: vitamin A deficiency. www.childinfo.org/eddb/vita_a/.

[B8] Unicef (2005). At a glance: Pakistan. http://www.unicef.org/infobycountry/pakistan_statistics.html.

[B9] WHO Global prevalence of vitamin A deficiency MDIS Working Paper # 2. Geneva: WHO, 1995..

[B10] Paracha PI, Jamil A, Northrop-Clewes CA, Thurnham DI (2000). Interpretation of vitamin A status in apparently healthy Pakistani children by using markers of subclinical infection. Am J Clin Nutr.

[B11] Molla A, Badruddin SH, Khurshid M, Molla AM, Rahaman FN, Durrani S, Suria A, Snyder JD, Hendricks K (1993). Vitamin A status of children in the urban slums of Karachi, Pakistan, assessed by clinical, dietary, and biochemical methods. Am J Trop Med Hyg.

[B12] Kasi PM, Gilani AI, Ahmad K, Janjua NZ (2004). Blinding trachoma: a disease of poverty. PLoS Med.

[B13] WHO Prevention of childhood blindness. Geneva: WHO, 1992.

[B14] Hughes BR, Wetton N, Collins M, Newton Bishop JA (1996). Health education about sun and skin cancer: language, ideas and perceptions of young children. Br J Dermatol.

[B15] Oakley A, Bendelow G, Barnes J, Buchanan M, Husain OA (1995). Health and cancer prevention: knowledge and beliefs of children and young people. Bmj.

[B16] Backett-Milburn K, McKie L (1999). A critical appraisal of the draw and write technique. Health Educ Res.

[B17] Williams DT, Wetton N, Moon A (1989). A Way In: Five Key Areas of Health Education. Health Education Authority, London..

[B18] McWhirter JM, Collins M, Bryant I, Wetton NM, Newton Bishop J (2000). Evaluating 'Safe in the Sun', a curriculum programme for primary schools. Health Educ Res.

[B19] Pion IA, Kopf AW, Hughes BR, Wetton NM, Collins M, Newton Bishop JA (1997). Teaching children about skin cancer: the draw-and-write technique as an evaluation tool. Pediatr Dermatol.

[B20] Rademaker M, Wyllie K, Collins M, Wetton N (1996). Primary school children's perceptions of the effects of sun on skin. Australas J Dermatol.

[B21] Sutter E, Foster A, Francis V (1989). Hanyane, a Village Struggles for Eye Health. Macmillan.

[B22] WHO Primary School Health Education Prototype Curriculum, WHO EMRO, Alexandria, Egypt. 1988.

[B23] Emerson PM, Cairncross S, Bailey RL, Mabey DC (2000). Review of the evidence base for the 'F' and 'E' components of the SAFE strategy for trachoma control. Trop Med Int Health.

[B24] Kumaresan JA, Mecaskey JW (2003). The global elimination of blinding trachoma: progress and promise. Am J Trop Med Hyg.

[B25] Mabey D, Bailey R (1999). Eradication of trachoma worldwide. Br J Ophthalmol.

[B26] Schachter J, Dawson CR (2002). Elimination of blinding trachoma. Curr Opin Infect Dis.

[B27] Pokharel GP, Negrel AD, Munoz SR, Ellwein LB (2000). Refractive Error Study in Children: results from Mechi Zone, Nepal. Am J Ophthalmol.

[B28] Limburg H, Vaidyanathan K, Dalal HP (1995). Cost-effective screening of schoolchildren for refractive errors. World Health Forum.

[B29] West ES, Schein OD (2005). Sunlight and age-related macular degeneration. Int Ophthalmol Clin.

[B30] Asbell PA, Dualan I, Mindel J, Brocks D, Ahmad M, Epstein S (2005). Age-related cataract. Lancet.

[B31] Awan AA, Khan T, Mohammad S, Arif AS (2002). Eclipse retinopathy: follow up of 36 cases after April 1995 solar eclipse in Pakistan. J Ayub Med Coll Abbottabad.

[B32] Bozin I (1967). [2 cases of macular burns after watching a solar eclipse]. Ann Ocul (Paris).

[B33] Rothkoff L, Kushelevsky A, Blumenthal M (1978). Solar retinopathy: visual prognosis in 20 cases. Isr J Med Sci.

[B34] Barr A, Baines PS, Desai P, MacEwen CJ (2000). Ocular sports injuries: the current picture. Br J Sports Med.

[B35] Dandona L, Dandona R, Srinivas M, John RK, McCarty CA, Rao GN (2000). Ocular trauma in an urban population in southern India: the Andhra Pradesh Eye Disease Study. Clin Experiment Ophthalmol.

[B36] Lithander J, Al Kindi H, Tonjum AM (1999). Loss of visual acuity due to eye injuries among 6292 school children in the Sultanate of Oman. Acta Ophthalmol Scand.

[B37] Serrano JC, Chalela P, Arias JD (2003). Epidemiology of childhood ocular trauma in a northeastern Colombian region. Arch Ophthalmol.

[B38] Goss DA (2000). Nearwork and myopia. Lancet.

[B39] Wong TY, Saw SM (2004). Issues and challenges for myopia research. Ann Acad Med Singapore.

[B40] al-Hazzaa SA, Krahn PM (1995). Kohl: a hazardous eyeliner. Int Ophthalmol.

[B41] Alkhawajah AM (1992). Alkohl use in Saudi Arabia. Extent of use and possible lead toxicity. Trop Geogr Med.

[B42] Hardy A, Walton R, Vaishnav R (2004). Composition of eye cosmetics (kohls) used in Cairo. Int J Environ Health Res.

[B43] Parry C, Eaton J (1991). Kohl: a lead-hazardous eye makeup from the Third World to the First World. Environ Health Perspect.

[B44] Canfield RL, Henderson CRJ, Cory-Slechta DA, Cox C, Jusko TA, Lanphear BP (2003). Intellectual impairment in children with blood lead concentrations below 10 microg per deciliter. N Engl J Med.

[B45] Canfield RL, Kreher DA, Cornwell C, Henderson CRJ (2003). Low-level lead exposure, executive functioning, and learning in early childhood. Neuropsychol Dev Cogn C Child Neuropsychol.

